# Dynamic assembly of the mRNA m^6^A methyltransferase complex is regulated by METTL3 phase separation

**DOI:** 10.1371/journal.pbio.3001535

**Published:** 2022-02-10

**Authors:** Dasol Han, Andrew P. Longhini, Xuemei Zhang, Vivian Hoang, Maxwell Z. Wilson, Kenneth S. Kosik

**Affiliations:** 1 Neuroscience Research Institute, University of California, Santa Barbara, California, United States of America; 2 Department of Molecular, Cellular and Developmental Biology, University of California, Santa Barbara, California, United States of America; Yale University, UNITED STATES

## Abstract

m^6^A methylation is the most abundant and reversible chemical modification on mRNA with approximately one-fourth of eukaryotic mRNAs harboring at least one m^6^A-modified base. The recruitment of the mRNA m^6^A methyltransferase writer complex to phase-separated nuclear speckles is likely to be crucial in its regulation; however, control over the activity of the complex remains unclear. Supported by our observation that a core catalytic subunit of the methyltransferase complex, METTL3, is endogenously colocalized within nuclear speckles as well as in noncolocalized puncta, we tracked the components of the complex with a Cry2-METTL3 fusion construct to disentangle key domains and interactions necessary for the phase separation of METTL3. METTL3 is capable of self-interaction and likely provides the multivalency to drive condensation. Condensates in cells necessarily contain myriad components, each with partition coefficients that establish an entropic barrier that can regulate entry into the condensate. In this regard, we found that, in contrast to the constitutive binding of METTL14 to METTL3 in both the diffuse and the dense phase, WTAP only interacts with METTL3 in dense phase and thereby distinguishes METTL3/METTL14 single complexes in the dilute phase from METTL3/METTL14 multicomponent condensates. Finally, control over METTL3/METTL14 condensation is determined by its small molecule cofactor, S-adenosylmethionine (SAM), which regulates conformations of two gate loops, and some cancer-associated mutations near gate loops can impair METTL3 condensation. Therefore, the link between SAM binding and the control of writer complex phase state suggests that the regulation of its phase state is a potentially critical facet of its functional regulation.

## Abstract 2

Approximately one-fourth of eukaryotic mRNAs harbor at least one m6A-modified base, but how is this regulated? This study shows that cells can use liquid-liquid phase separation to regulate dynamic assembly of the mRNA m6A methyltransferase complex (METTL3/METTL14/WTAP), with stoichiometries that depend on condensate partitioning in a substrate binding-dependent manner.

## Introduction

m^6^A is the most abundant posttranscriptional modification on mRNA [1], which affects myriad biological processes such as embryonic stem cell differentiation [2–4], brain development [5], synaptic plasticity [6,7], hematopoiesis [8], and cancer [9,10]. m^6^A-specific proteins of the YTH domain family (readers) bind modified RNAs to exert m^6^A-dependent regulation of target transcripts, affecting pre-mRNA splicing [11], translation initiation [12,13], stress granule localization [9], and RNA export, stability, and decay [12,14,15]. mRNA m^6^A modification is mediated by methyltransferase (writer) complex components, methyltransferase-like 3 (METTL3), methyltransferase-like 14 (METTL14), and Wilms tumor suppressor-1–associated protein (WTAP) as characterized by multiple groups elucidating mechanistic insights based on crystal structures and in vitro experiments [3,16–19]. METTL3 and METTL14 constitutively interact to form a heterodimer via their methyltransferase domains (MTDs) thereby providing a binding site for RNA, the acceptor substrate [3,16–18,20]. On the other hand, the donor substrate S-adenosylmethionine (SAM) can only bind METTL3 because the SAM binding pocket of METTL14 has degenerated [16–18]. Despite the indispensability of WTAP on m^6^A modification [21], its interaction dynamics in the writer complex is relatively unexplored compared to METTL3/14 complex.

Membraneless liquid compartments, such as stress granules and processing bodies in cytosol [22] and nucleoli [23], Cajal bodies [24], and nuclear speckles [25] in nucleus have roles in numerous cellular functions. Often, they are abundant in RNA and affect transcriptomic changes [26,27]. For example, nuclear speckles are important RNA containing membraneless organelles that function as sites for RNA splicing and RNA m^6^A methylation. Liquid–liquid phase separation (LLPS) of these compartments selects biomolecules to become concentrated presumably to implement their functions [28,29]. LLPS is often multiphasic with key molecules selectively partitioning into different layers of individual droplets as seen in nuclear speckles [30]. LLPS of biomolecules requires multivalent interactions, which are mediated by scaffold proteins with tandem repeat binding sites for other partners [31] or proteins with intrinsically disordered regions (IDRs, or low-complexity domains), often in concert with nucleic acids [31,32]. Recently, these findings and studies have been facilitated by optogenetic tools that regulate protein clustering by light stimulation within the live-cell context. The use of optogenetic tools, such as photolyase homology region of *Arabidopsis thaliana* Cry2, lowers the free energy for LLPS of proteins predisposed to phase separate and endows precisely tunable switches for biomolecular interactions that mediate LLPS [33,34]. Key biomolecular interactions are probed rapidly and reliably by artificially linking full-length, as well as mutant and truncated proteins to the Cry2 system and interrogating the system with light-induced clustering.

m^6^A-methylated mRNAs can be recognized by YTH domain containing family proteins (YTHDF1-3) and affected according to the cellular context [11,12,15,35]. Recent studies have shown that the YTHDFs can undergo LLPS via their IDRs and the phase separation potential can be further facilitated by multiple m^6^A modifications on RNA, suggesting an important role of LLPS on cellular transcriptomic regulation by RNA modification [36–38]. The potential role of LLPS in the mammalian m^6^A writer complex, which has been proposed to interact with nuclear speckles has not been explored. Here, we used immunocytochemistry (ICC) to show that the endogenous writer complex colocalizes with a recognizable phase separated compartment, nuclear speckles. With optogenetic and fluorescent lifetime imaging microscopy (FLIM) tools, we demonstrate that cells can utilize LLPS to regulate dynamic assembly of mRNA m^6^A methyltransferase complex (METTL3/METTL14/WTAP) with stoichiometries that depend on condensate partitioning in a substrate binding–dependent manner.

## Results

### Compartmentation of endogenous METTL3 in the nucleus

In an effort to explore the role of LLPS in RNA methylation, we explored the association of METTL3, a catalytic subunit of the m^6^A methyltransferase complex, with nuclear speckles. METTL3 is known to colocalize with nuclear speckle markers as bright punctate densities [20,21,39]. With higher resolution images, METTL3 shows a layered distribution relative the nuclear speckle marker pSC35 (S1A Fig). We characterized the radial distribution of METTL3 around centroids of the nuclear speckle marker pSC35. A clear spike in the METTL3 distribution is seen around the periphery of the pSC35 signal (S1A Fig). This layered organization within the nuclear speckle is consistent with other components of these organelles, specifically spliceosomes, RNA introns, and scaffolding proteins [30]. METTL3 also labeled similar puncta unassociated with nuclear speckles, but with patterns typical of phase separated entities. The multiphasic behavior of METTL3 and its complex compartmentation prompted further experiments into the molecular determinants of METTL3 phase separation.

### METTL3 undergoes optogenetically inducible liquid–liquid phase separation (LLPS)

**Fig 1:**
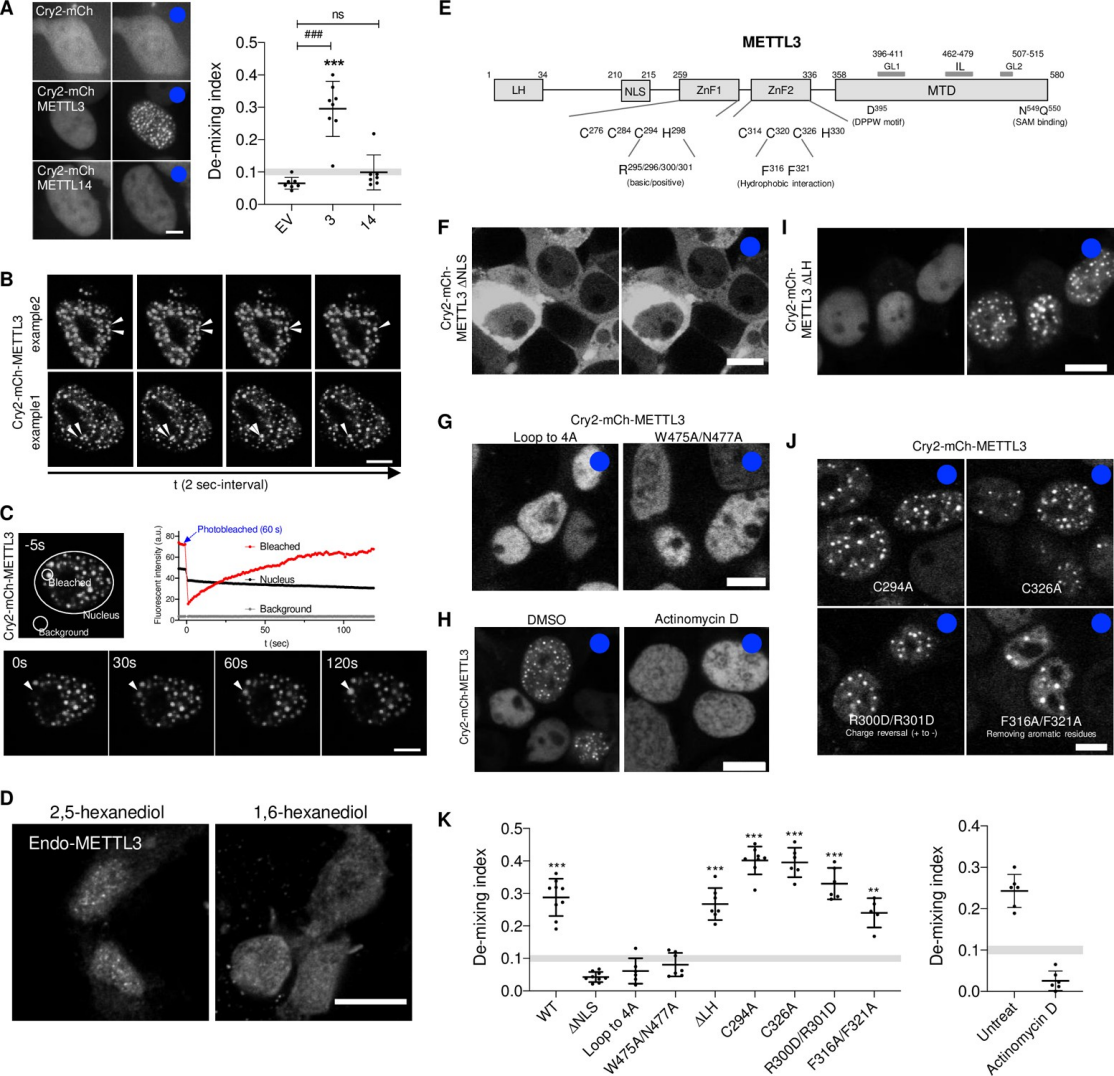
LLPS of Cry2-mCherry-METTL3. (A) (Left) Representative confocal images of HEK293T expressing Cry2-mCherry constructs before and after (blue dots) blue light (488 nm) stimulation. (Right) Demixing index at 300 seconds of blue light stimulation was quantified by CV across the nucleus. Gray area (0.09~0.11) is set as threshold of condensate formation. See S1C Fig. Scale bar = 5 μm, Student *t* test used to compare between samples ###, *P* < 0.001; ns, nonsignificant. One-sample *t* test used to compare demixing index above gray area to hypothetical threshold (0.11) ***, *P* < 0.001, error bars represent mean ± SD (*n =* 7 for EV and METTL3 and *n* = 8 for METTL14). EV (Cry2-mCherry); 3, Cry2-mCh-METTL3; and 14, Cry2-mCh-METTL14. (B) Two representative time series images of Cry2-mCherry-METTL3 showing fusion events. White arrowheads track the fusion of 2 condensates. Time interval = 2 seconds. Scale bar = 5 μm. (C) Representative time series images from 3 independent FRAP experiments using Cry2-mCh-METTL3 expressing HEK293T cells (top images) and quantitation of fluorescent intensity (bottom plot). Arrowhead tracks bleached area. Fluorescent intensity was measured using ImageJ software. Scale bar = 5 μm. (D) Representative images of endogenous METTL3 in HeLa cells treated with 5% 2,5- or 1,6-hexanediol for 5 minutes. Scale bar = 20 μm. (E) Schematic diagram of METTL3 domains. (F) Representative images showing localization and protein distribution of Cry2-mCh-METTL3 ΔNLS before (left) and after (right, blue dot) blue light stimulation. Scale bar = 10 μm. (G) Light stimulation of METTL14 binding–deficient mutants of Cry2-mCh-METTL3. Blue dot indicates light stimulation. Scale bar = 10 μm. (H) Light-inducible condensate formation of Cry2-mCh-METTL3 (wild-type) with Actinomycin D (2 μg/ml) or vehicle (DMSO). Blue dots indicate light stimulation. Scale bar = 10 μm. (I) Light stimulation of Cry2-mCh-METTL3 ΔLH mutant. Blue dot indicates light stimulation. Scale bar = 10 μm. (J) Representative images showing protein distribution of Cry2-mCh-METTL3 ZnF1/2 mutant after (blue dot) blue light stimulation. Scale bar = 10 μm. (K) Demixing index at 300 seconds of blue light stimulation was quantified with mutant constructs (Fig 1F, 1G, 1I, and 1J, left) and wild type with/without Actinomycin D (Fig 1H, right). Gray area (0.09~0.11) is set as threshold of condensate formation. One-sample *t* test used to compare demixing index above gray area to hypothetical threshold (0.11) **, *P* < 0.01; ***, *P* < 0.001, error bars represent mean ± SD (*n =* 9 for WT and ΔNLS; *n* = 8 for C294A; *n* = 7 for W475A/N477A and ΔLH; *n* = 6 for loop to 4A, C326A, R300D/R301D, DMSO, and Actinomycin D; and *n* = 5 for F316A/F321A). The underlying data for the graphs presented can be found in S1 Values For Plots. a.u., arbitrary unit; CV, coefficient of variation; EV, empty vector; FRAP, fluorescence recovery after photobleaching; GL, gate loop; IL, interface loop; LH, leader helix; LLPS, liquid–liquid phase separation; MTD, methyltransferase domain; METTL3, methyltransferase-like 3; METTL14, methyltransferase-like 14; NLS, nuclear localization signal; ZnF, zinc-finger motif.

Based upon the above observations and previous studies showing that many RNA binding proteins (RBPs) undergo LLPS via multivalent interactions [33,40–42], we hypothesized that the mRNA m^6^A methyltransferase complex undergoes regulated dynamic phase transition. The core components of m^6^A mRNA methyltransferase complex are METTL3 and METTL14, which are hypothesized to constitutively bind each other and form a heterodimer [3]. To test if these core subunits are independently capable of LLPS, METTL3 and METTL14 were fused to the photolyase homology region of *A*. *thaliana* Cry2, a light-activatable self-associating protein and mCherry [33,43] (S1B Fig). In the absence of light activation, Cry2-mCh-METTL14 appears to undergo rapid degradation when expressed by itself but is readily detectable when coexpressed with METTL3, suggesting that METTL3 can stabilize METTL14. Conversely, Cry2-mCh-METTL3 is readily detectable when expressed by itself (S1C Fig). Light activation of the Cry2-mCh-METTL3 full-length construct results in the formation of round METTL3 condensates within seconds of blue light exposure (Figs 1A and S1D). However, light activation of Cry2-mCh-METTL14 did not induce condensation (Fig 1A) even when stabilized by coexpression of FLAG-METTL3 (S1E Fig), indicating that METTL3 but not METTL14 is capable of light-inducible LLPS. Cry2-mCh-METTL3 condensates displayed liquid properties as shown by fusion (Fig 1B) and fluorescence recovery after photobleaching (FRAP) assays (Fig 1C). Endogenous METTL3 is stained in a punctate pattern, which is dissolved by 1,6-hexanediol treatment [44], indicating the LLPS nature of the structure (Fig 1D).

### Nascent RNA is essential for METTL3 opto-condensate formation

As METTL3 does not contain a large IDR (S1F Fig) found in some RBPs reported to undergo LLPS [33,41], we sought to investigate the roles of the METTL3 structural and functional domains (Fig 1E) with regard to its LLPS behavior. Recent structural studies acted as a rational design of our mutant METTL3 constructs [16–19]. Deleting the nuclear localization signal (NLS) completely blocked condensate formation, indicating that nuclear environment is required for LLPS of METTL3 (Fig 1F). We rationalized this observation by the fact that m^6^A writer complex components, such as METTL14, WTAP, and nascent RNA, are largely missing in the cytoplasm.

Interaction between METTL3 and METTL14 generates a positively charged groove where mRNAs bind [18]. To test if METTL14 and/or the RNA binding ability of METTL3 is required for the phase separation, we disrupted the METTL14 binding surface of Cry2-mCh-METTL3 by substituting either the whole (residues 462 to 479) or the core residues in the interface loop of METTL3 with alanines (loop to 4A and W475A/N477A). These mutants failed to form condensates indicating that an intact RNA binding groove in the interface loop is necessary for the phase separation of METTL3 (Fig 1G). We next asked if the existence of nascent mRNA molecules is itself important. Because m^6^A modification on mRNA occurs cotranscriptionally [45], cells were treated with actinomycin D for 4 hours to inhibit transcription and remove nascent RNA in the nucleus. This resulted in a complete absence of METTL3 phase separation (Fig 1H), indicating the necessity of a nascent acceptor substrate RNA. A previous report showed that RNase treatment led to an exclusion of METTL3 from nuclear speckles, further supporting our observations [21].

The leader helix (LH) and 2 type-CCCH Zinc finger (ZnF) domains, which are responsible for WTAP binding and sequence specificity of RNA substrates, respectively [19,46], were dispensable for LLPS revealed by LH deletion- (ΔLH), ZnF disrupting- (C294A for ZnF1, C326A for ZnF2), charge reversal- (R300D/R301D), and aromatic ring-removing (F316A/F321A) mutants (Figs 1I and 1J and S2A). Overall, the nucleus provides a favorable environment to the LLPS of METTL3 by concentrating key components of the methyltransferase complex: METTL14 and nascent RNA (Fig 1K).

### METTL3 self-interacts, providing multivalency for LLPS of mRNA m^6^A methyltransferase complex

**Fig 2:**
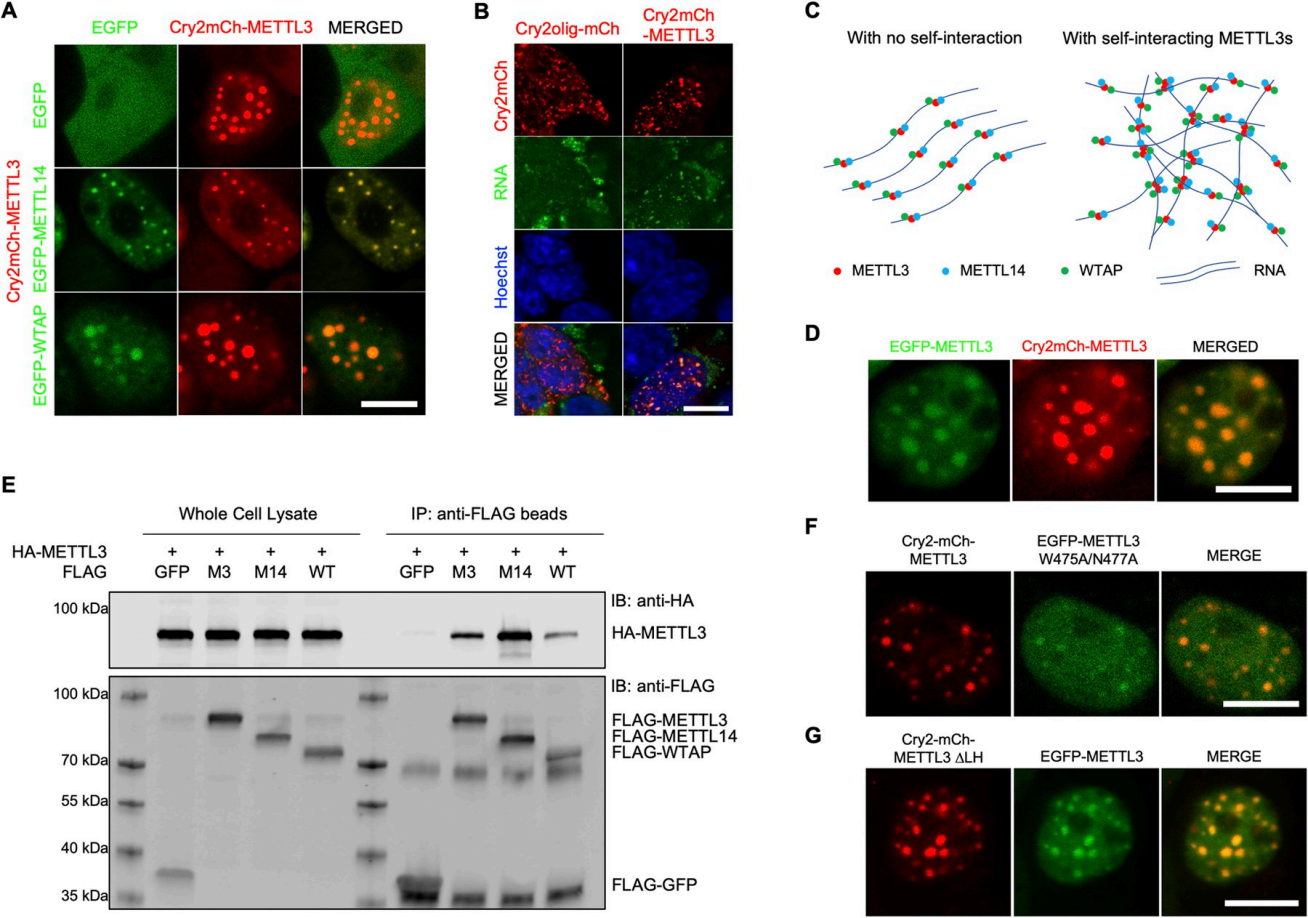
Self-interaction of METTL3 provides multivalency for LLPS of the writer complex. (A) Representative images showing localization of EGFP control, EGFP-METTL14 or EGFP-WTAP after Cry2-mCh-METTL3 condensates formation by light stimulation. Scale bar = 10 μm. (B) Colocalization of RNA (SYTO RNAselect live cell stain) and Cry2-mCh-METTL3 condensates. Cry2olig-mCh (Addgene #60032) is used as light-inducible oligomerization control. Scale bar = 10 μm. (C) Hypothetical models of intermolecular interactions mRNA m^6^A methyltransferase complex with (right) or without (left) self-interacting ability of METTL3. (D) Representative images showing localization of EGFP-METTL3 after Cry2-mCh-METTL3 condensates formation by light stimulation. (E) Co-immunoprecipitation assay with HEK293T coexpressing HA-METTL3 and FLAG-tagged METTL3 (M3), METTL14 (M14), or WTAP (WT). (F) Recruitment of METTL14 binding–deficient mutant METTL3 into Cry2-mCh-METTL3 liquid condensates. Scale bar = 10 μm. (G) Recruitment of METTL3 into Cyr2-mCh-METTL3 ΔLH mutant liquid condensates. Scale bar = 10 μm. LLPS, liquid–liquid phase separation; METTL3, methyltransferase-like 3; METTL14, methyltransferase-like 14; WTAP, Wilms tumor suppressor-1–associated protein.

METTL3 interacts with METTL14 and WTAP for efficient methyltransferase activity [16–18,20]. To test if optogenetically inducible METTL3 condensates contain intact methyltransferase complexes, EGFP-fused METTL14 or WTAP were coexpressed with Cry2-mCh-METTL3 (S1B Fig). Upon induction of METTL3 condensates with blue light, METTL14 and WTAP are readily recruited into the condensates (Fig 2A). Cry2-METTL3 condensates also contained RNA as shown by SYTO RNAselect live cell RNA staining (Fig 2B). This was not Cry2 domain driven, as confirmed by light-inducible Cry2olig aggregates [34] that do not colocalize with RNA (Fig 2B).

LLPS requires multivalent interactions [29,31]. A variety of RBPs that undergo LLPS harbor IDRs that contribute multivalent interactions [32,33,41]. In METTL3, however, the only potential IDR was the N-terminally located LH domain (S1F Fig), where WTAP binds [46]. However, METTL3 ΔLH still formed condensates without WTAP recruitment (Figs 1I and S2A), indicating that METTL3 LLPS is not IDR driven. Although a single RNA molecule could have multiple m^6^A modification sites [47], it is insufficient to explain multivalent interaction and LLPS because the valency of this system is only one, as the RNA would not have a bridge for crosslinking (Fig 2C). To address this unanswered question, we hypothesized that METTL3 builds homodimers that provide the necessary multivalency (Fig 2C). Indeed, EGFP-METTL3 was also recruited into Cry2-METTL3 liquid condensates (Fig 2D). By co-immunoprecipitation assay, we confirmed the interaction between differentially tagged METTL3 recombinant proteins (Figs 2E and S3A). The interaction between METTL3 proteins was not mediated by the interface loop/RNA binding via METTL14 interaction as shown by recruitment of METTL3 W475A/N477A, which lacks LLPS ability when fused to Cry2, but does condense with wild-type METTL3 (Fig 2F). Furthermore, METTL3 ΔLH condensates also recruited EGFP-METTL3 (wild type), suggesting a WTAP-independent interaction (Fig 2G). These data demonstrate that the self-interacting ability of METTL3 provide the necessary multivalency for LLPS of mRNA m^6^A methyltransferase complex.

### Phase-dependent dynamic assembly of mRNA m^6^A methyltransferase complex

**Fig 3:**
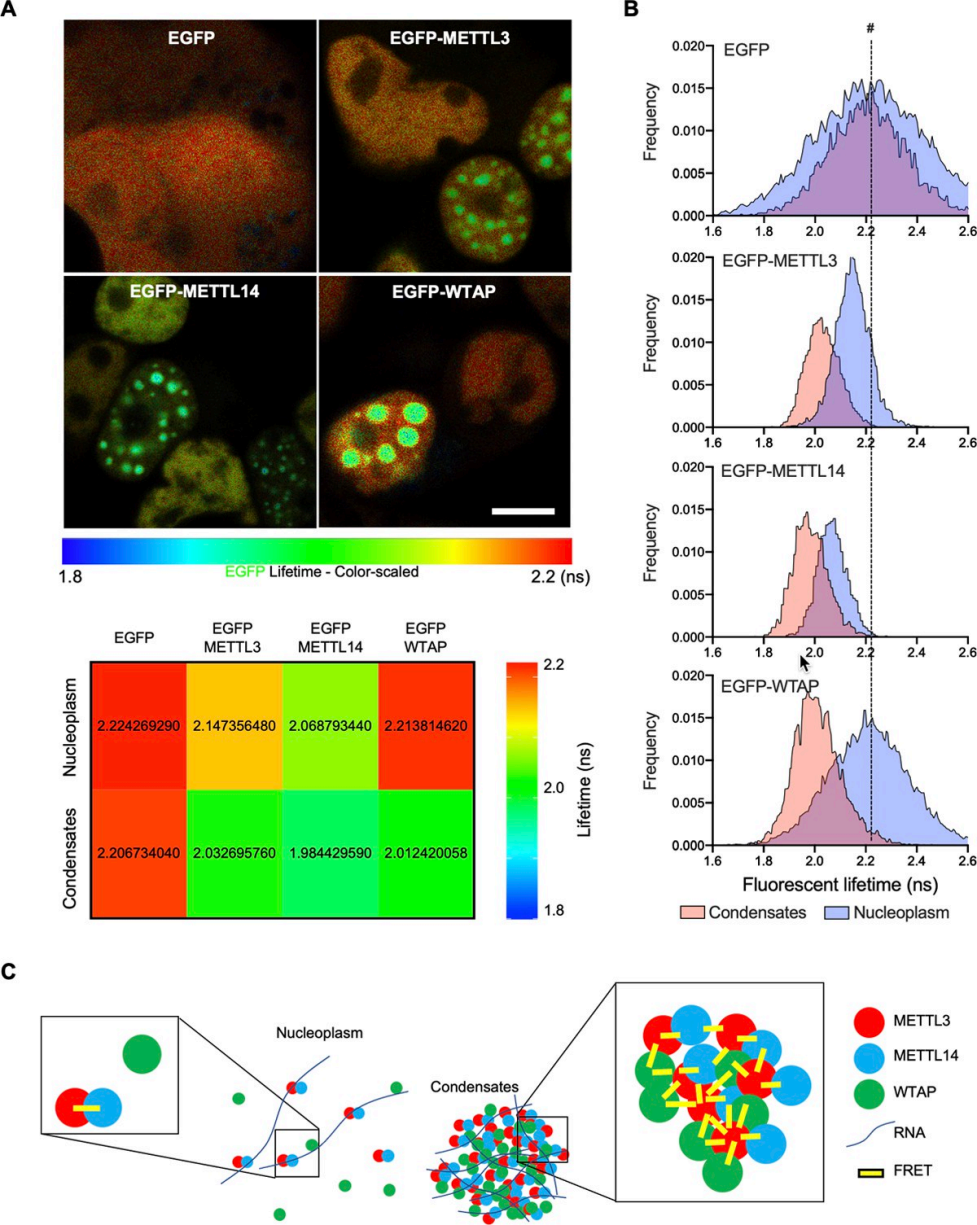
FLIM-FRET reveals the phase-dependent differential interactions of the m^6^A writer complex components. (A) (Top) In vivo EGFP lifetime maps of EGFP control, EGFP-METTL3, EGFP-METTL14, and EGFP-WTAP. Scale bar = 10 μm. (Bottom) Color heatmap representing mean fluorescent lifetime values (ns) measured from nucleoplasm without condensates (Nucleoplasm) or inside condensates (Condensates) of each EGFP-fused protein. (B) Histograms showing fluorescent lifetime distribution (>300,000 photons) measured from nucleoplasm without condensates (Nucleoplasm) or inside condensates (Condensates) of each EGFP-fused protein. # and vertical dashed line indicate control EGFP lifetime in nucleoplasm without condensates. (C) Schematic diagram describing the intermolecular distances and FRET activity based on observations. The underlying data for the graphs presented can be found in S1 Values For Plots. FLIM-FRET, fluorescent lifetime imaging microscopy–Förster resonance energy transfer; METTL3, methyltransferase-like 3; METTL14, methyltransferase-like 14; WTAP, Wilms tumor suppressor-1–associated protein.

We next probed the interactions among the components of the m^6^A writer complex. Biomolecular condensates allow a selective set of biomolecules across the phase boundary to assemble multicomponent organelle capable of biological activities [29,32]. To measure the interactions of the m^6^A writer complex components in condensates, we conducted fluorescent lifetime imaging microscopy–Förster resonance energy transfer (FLIM-FRET) experiments. Limitations related to photobleaching during live-cell imaging with intensity-based FRET measurements prompted the use of an intensity-independent FRET measurement [48] because fluorescent lifetime is an intrinsic property of the donor fluorophore, which is independent of its concentration and, therefore, not affected by photobleaching. During FLIM-FRET, the fluorescent lifetime of the donor fluorophore is monitored, and if FRET occurs, the measured lifetime undergoes a characteristic shift to lower values. In all experiments described below Cry2-METTL3 (mCherry) acted as an acceptor for fusion proteins to EGFP. In accordance with previous studies that showed constitutive interaction between METTL3 and METTL14 [16–18,20], when compared to EGFP only control, EGFP-METTL14 lifetimes were lowered (i.e., higher FRET activity) in the bulk nucleoplasm (2.22 ± 0.226 ns for EGFP versus 2.07 ± 0.058 ns for EGFP-METTL14) (Fig 3A and 3B). After condensate formation, EGFP-METTL14 lifetimes underwent a further drop (1.98 ± 0.069 ns) indicative of closer contact between the donor and acceptor fluorophores (Fig 3A and 3B). Consistent with the observation of METTL3 self-interaction (Fig 2D and 2E), EGFP-METTL3 lifetimes were shorter in condensates (2.03 ± 0.066 ns) when compared to that in bulk nucleoplasm (2.15 ± 0.071 ns). Given that the Förster distance (R0, the distance between 2 fluorophores leading to an energy transfer) between EGFP and mCherry is 5.24 ± 0.10 nm [49], this result further supported direct interaction between METTL3 proteins. Interestingly, the well-characterized METTL3-interacting protein WTAP [21,46] showed no FRET activity in the bulk nucleoplasm (2.22 ± 0.159 ns). However, upon condensate formation, FRET between EGFP-WTAP and METTL3 (mCherry) was observed (2.01 ± 0.095 ns for EGFP-WTAP) (Fig 3A and 3B). Altogether, these data suggest a phase-dependent differential multicomponent composition of mRNA m^6^A methyltransferase complex (Fig 3C).

### Donor substrate-dependent conformations of 2 gate loops in the catalytic site of METTL3 determine LLPS behavior

**Fig 4:**
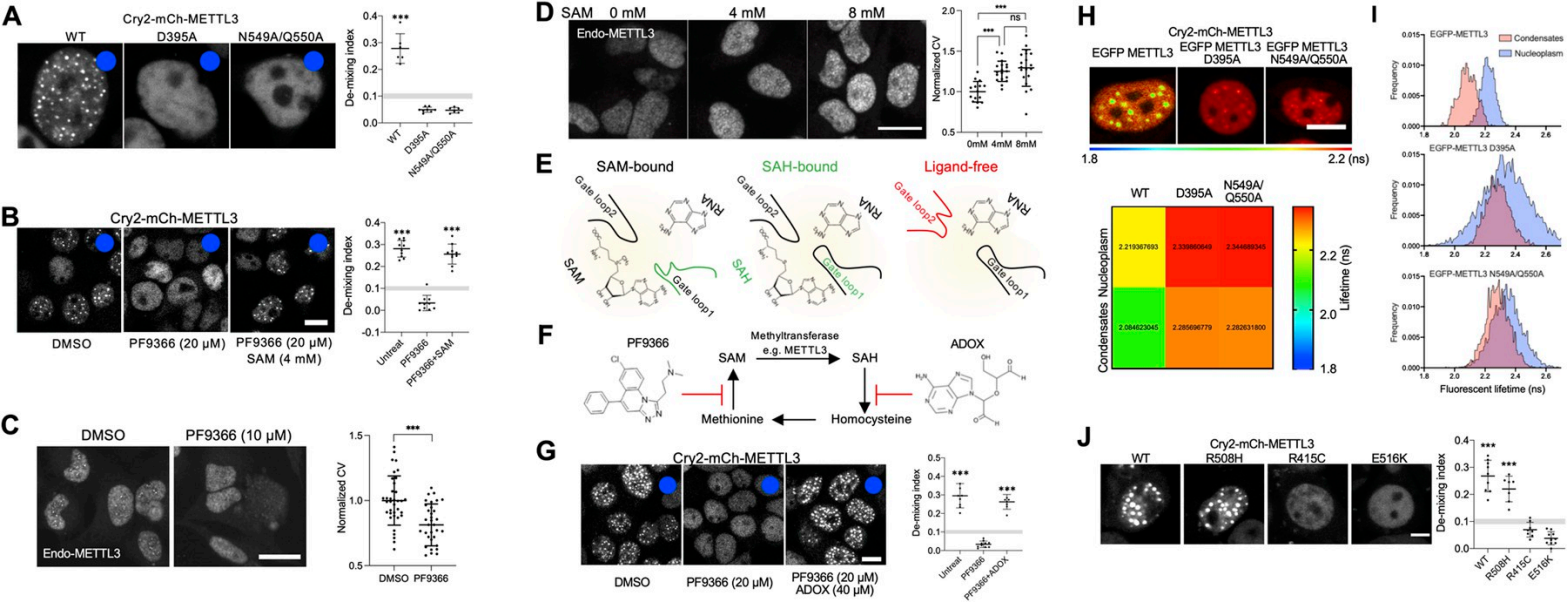
Ligand binding states determine METTL3 phase transition. (A) Representative images (left) and quantitation (right) representing demixing of Cry2-mCh-METTL3 D395A (catalytic motif, SAM binding pocket) or N549A/Q550A (SAM binding pocket) mutants after (blue dot) blue light stimulation. Scale bar = 10 μm. Gray area (0.09~0.11) is set as threshold of condensate formation. One-sample *t* test used to compare demixing index above gray area to hypothetical threshold (0.11) ***, *P* < 0.001, error bars represent mean ± SD (*n =* 8 for D395A and *n* = 6 for WT and N549A/Q550A). (B) Representative images (left) and quantitation (right) of effect of SAM depletion from the cells on light-inducible Cry2-mCh-METTL3 condensate formation. PF9366 is pretreated for 4 hours before blue light stimulation. SAM is added 3 hours after PF9366 treatment in presence of PF9366. Scale bar = 10 μm. Gray area (0.09~0.11) is set as threshold of condesate formation. One-sample *t* test used to compare demixing index above gray area to hypothetical threshold (0.11) ***, *P* < 0.001, error bars represent mean ± SD (*n =* 10). (C) Representative images (left) and quantitation (right) representing demixing of endogenous METTL3 at 4 hours after PF9366 treatment. Scale bar = 20 μm. Student *t* test, ***, *P* < 0.001, error bars represent mean ± SD (*n* = 36 for DMSO, *n* = 34 for PF9366). (D) Representative images (left) and quantitation (right) representing demixing of endogenous METTL3 at 15 minutes after SAM treatment. Scale bar = 20 μm. One-way ANOVA, ***, *P* < 0.001, error bars represent mean ± SD (*n* = 15 for 0 mM, *n* = 18 for 4 mM, and *n* = 19 for 8 mM). (E) Schematic diagram describing 3 gate loops’ conformations depending on ligand bound states of SAM binding pocket in METTL3. (F) Simplified SAM cycle and chemical inhibitors, PF9366 and ADOX, which block SAM synthesis and SAH hydrolysis, respectively. (G) Effects of small molecule inhibition of SAM cycle on light-inducible Cry2-mCh-METTL3 condensate formation. Scale bar = 10 μm. Gray area (0.09~0.11) is set as threshold of condensate formation. One-sample *t* test used to compare demixing index above gray area to hypothetical threshold (0.11) ***, *P* < 0.001, error bars represent mean ± SD (*n =* 8 for PF9366+ADOX, *n* = 9 for Untreat, and *n* = 10 for PF9366). (H) (Top) In vivo EGFP lifetime map of EGFP-METTL3 (wild-type and SAM binding mutants) in HEK293T with light-inducible METTL3 condensates. Scale bar = 10 μm. (Bottom) Color heatmap representing mean fluorescent lifetime values (ns) measured from nucleoplasm without condensates (Nucleoplasm) or inside condensates (Condensates) of each EGFP-fused protein. (I) Histograms showing fluorescent lifetime distribution (>300,000 photons) measured from nucleoplasm without condensates (Nucleoplasm) or inside condensates (Condensates) of each EGFP-fused protein. (J) Representative images (left) and quantitation (right) representing demixing of Cry2-mCh-METTL3 cancer mutants after blue light stimulation. Scale bar = 5 μm. Gray area (0.09~0.11) is set as threshold of condensate formation. One-sample *t* test used to compare demixing index above gray area to hypothetical threshold (0.11) ***, *P* < 0.001, error bars represent mean ± SD (*n* = 9 for WT, *n* = 7 for R508H, *n* = 8 for R415C, and *n* = 10 for E516K). The underlying data for the graphs presented can be found in S1 Values For Plots. METTL3, methyltransferase-like 3; SAH, S-adenosylhomocysteine; SAM, S-adenosylmethionine.

Given that only METTL3, but not METTL14, underwent LLPS, we next focused on their structural difference. In a mRNA m^6^A writer complex, SAM forms multiple hydrogen bonds with adjacent amino acids in SAM binding pocket of METTL3, whereas the SAM binding pocket in METTL14 is degenerate [16–18]. To test if the donor substrate SAM is required for LLPS, we introduced mutations at SAM binding pocket of Cry2-mCh-METTL3 (D395A, N549A/Q550A). All mutants failed to form condensates while retaining interactions with both METTL3 and METTL14 (Figs 4A and S4A and S4B). These data were highly suggestive that SAM binding could be a crucial factor for phase transition. To exclude the possibility that conformational change caused by the mutation rather than SAM binding affected the LLPS behavior, we removed SAM by treating cells with PF9366, a specific potent inhibitor of SAM synthetase MAT2A (Fig 4F) [50]. Treatment with PF9366 for 4 hours completely prevented Cry2-METTL3 from phase separating (Fig 4B), confirming the necessity of SAM for LLPS. Supplying SAM into PF9366-treated cells rescued the blockage of condensate formation (Fig 4B), ruling out the possibility that the observation was due to a side effect of PF9366. Furthermore, the demixed state of endogenous METTL3 was affected by deprivation or addition of SAM (Fig 4C and 4D). To our knowledge, this is the first demonstration of an endogenous small molecule cofactor exerting control over LLPS behavior of its enzyme.

Wang and colleagues [17] suggested that the recognition of the adenosine substrate of METTL3 might depend on the ligand binding states of the SAM binding pocket, which, in turn, determines the conformational states of 2 gate loops: gate loop 1 (396 to 411) and gate loop 2 (507 to 515) (Figs 1E and 4E). Gate loop 1 is flipped outwards in the SAM-bound state compared to that in the S-adenosylhomocysteine (SAH)-bound or ligand-free states. Gate loop 2 closes the binding pocket when the SAM or SAH is bound, and undergoes a conformational change in the ligand-free state (Fig 4E) [17]. This led to the hypothesis that the ligand-binding states of SAM binding pocket in METTL3 could differentially affect the phase separation behavior of Cry2-METTL3. Although the SAH hydrolase reaction is reversible and presents a thermodynamic equilibrium that favors SAH synthesis [51], whether the SAM-binding pockets of overexpressed Cry2-METTL3 with PF9366 treatment are SAH-bound or ligand-free states remains unclear. To test if SAH-bound state is capable of LLPS, we pharmaceutically blocked SAH hydrolase activity with adenosine dialdehyde (ADOX) [52] (Fig 4F), causing an accumulation of SAH and the dominant occupation of SAH in the SAM binding pocket together with PF9366 treatment. ADOX/PF9366-treated cells still generated Cry2-METTL3 condensates (Fig 4G), indicating that the SAH-bound state of METTL3 is capable of phase transition. Taken together, among the 3 possible ligand binding states of Cry2-METTL3, SAM-bound (untreated or PF9366+SAM), SAH-bound (PF9366+ADOX), and ligand-free (PF9366), only ligand-free state inhibited condensate formation. Finally, we examined EGFP-fused SAM binding–deficient mutants (D395A and N549A/Q550A) in the presence of Cry2-METTL3 (wild-type) condensates. Interestingly, while retaining the ability to incorporate into condensates, these mutants showed dramatically reduced FRET activities (Fig 4H and 4I), implying that perturbed SAM binding affected the molecular arrangement inside condensates. These data collectively suggest the regulatory role of the ligand binding states on molecular interactions of mRNA m^6^A writer complex components and, thus, LLPS.

### LLPS behavior of METTL3 cancer mutants

Finally, we searched for relatively frequent METTL3 mutations in various cancers using The Cancer Genome Atlas (TCGA; https://www.cancer.gov/tcga). We picked a few mutations: R508H (found in corpus uteri, uterus), R415C (colon and stomach), and E516K (bladder cancer) to test on our Cry2 LLPS system. Interestingly, R415C and E516K mutants failed to phase separate upon Cry2 fusion and blue light illumination (Fig 4J). It is noteworthy that these mutations are located near the gate loops (gate loop 1: 396 to 411 and gate loop 2: 507 to 515). Although these observations are not direct evidence to relate LLPS ability to tumorigenesis, it will be of interest to investigate the relationship between phase behavior and normal function of METTL3, and the causative role of specific mutants on LLPS.

## Discussion

Using endogenous staining, optogenetic and FLIM-FRET tools, the current study shows that METTL3 undergoes phase separation in nuclei. Specifically, in an optogenetically inducible system, METTL3 phase separates in a substrate-dependent manner to dynamically regulate the assembly of the METTL3/METTL14/WTAP writer complex. Phase separation of the catalytic subunit METTL3 is regulated by the functionally conserved SAM-binding pocket in METTL3, which is degenerate in METTL14 [16–18]. Indeed, mutagenesis and chemical approaches revealed that the ligand binding state of the SAM binding pocket determines METTL3 phase separation behavior (Fig 4). Based on the previous observation that METTL3/METTL14 methyltransferase complex can form a dimer of dimer [20], we demonstrated the self-interaction of METTL3 can act as a multivalent scaffold for LLPS (Fig 2D and 2E).

The core components of m^6^A mRNA methyltransferase complex, METTL3/14 and WTAP, have been identified and characterized by multiple groups [3,20,21,39,53]. The mode of action of the METTL3 and METTL14 heterodimer during catalysis is relatively well characterized [16–18]. However, the interaction dynamics of WTAP with METTL3/METTL14 dimer remained unclear. Zhao’s group reported that METTL3 and METTL14 forms a stable heterodimer in a stoichiometry of 1:1 [3]. On the other hand, WTAP binds to the METTL3/14 heterodimer with much lower stoichiometry [20]. In this study, FLIM-FRET analysis demonstrated that METT3 differentially interacts with METTL14 versus WTAP. METTL14 constitutively binds METTL3 in both the dilute and the dense phases, whereas WTAP only shows FRET in METTL3 condensates (Fig 3A and 3B). The relative strengths of these multiple interactions strongly suggests that they determine the partition coefficients of the condensates, thus establishing an entropic barrier that can regulate entry into the condensate. Our observations might explain why only a certain proportion of WTAP molecules were bound to METTL3/14 complex in the previous study [20]. Furthermore, given that WTAP can regulate the localization of METTL3 and METTL14 to nuclear speckles [21], the phase-dependent dynamic interaction of WTAP shown in Fig 4 (FLIM-FRET) could provide a mechanism by which METTL3 enzymatic activity is regulated. Specifically, WTAP may be responsible for regulating the proportions of endogenous METTL3 droplets that are colocalized versus distinct from nuclear speckles.

The association of m^6^A mRNA methyltransferase complex with nuclear speckles has been well documented, but their relationship is more nuanced than previously reported. Specifically, METTL3 is layered within the nuclear speckle (S1A Fig), reminiscent of other components within membraneless organelles [30,54]. Multiphasic droplet systems that share interfaces but do not completely mix present an opportunity for subcompartmentalizing related, but distinct enzymatic processes such as RNA methylation and RNA splicing.

METTL3 phase separation occurred despite the lack of tandem repeat domain or large IDRs that are frequently found in RBPs [31,33,41] undergoing LLPS (S1F Fig). Thus, noncanonical determinants of LLPS may exist in METTL3 [31]. Mutagenesis and chemical approaches showed that donor substrate binding states determine phase transition of its enzyme complex (Fig 4A, 4B and 4G). Thus, in addition to the binding of nascent RNA as a regulator of phase separation (Fig 1H), the small molecule SAM (or SAH) can also mediate METTL3-driven multivalent interaction (Fig 4).

The phase separating properties of m^6^A-modified mRNA has been recently reported. m^6^A reader proteins (YTHDF1, 2, and 3) undergo LLPS that is enhanced by mRNAs with multiple m^6^A residues, enabling selective shuttling of target mRNAs to P-bodies, stress granules, or neuronal RNA granules [37,38]. More recently, Fu and colleagues reported that the m^6^A-binding YTHDF proteins promote stress granule formation by reducing the activation energy barrier and critical size for stress granule formation [36]. In addition, LLPS of MTA (the plant homolog of METTL3) was independently observed in a report published during the revision of the current study [55]. The authors showed that Cry photoreceptors in *A*. *thaliana* directly bind MTA and undergo light-driven LLPS to condense the m^6^A writer complex in vivo. This report accords with our hypothesis that m^6^A writer proteins undergo LLPS to increase local concentration and enzyme activity. However, Cry1 and Cry2 are absent in the mammalian genome, raising a question: How is the condensation of the m^6^A writer complex induced without photoreceptors? In the current study, we suggest another layer of LLPS regulation via substrate binding that conformationally changes METTL3 to enable multivalent interactions. Indeed, the concentration of SAM determined endogenous METTL3 condensation without Cry2 (Fig 4C and 4D). Our data also showed the complete disruption of LLPS competency with Cry2-METTL3 either by point mutations or by depletion of substrates, suggesting that Cry2 may be necessary but not sufficient for light-activated LLPS in *A*. *thaliana*. In addition, our fluorescence lifetime measurements show phase-dependent molecular arrangement in the condensed m^6^A writer complex (Fig 3), adding another potential mechanism of activity regulation. These observations collectively describe how cells utilize and control the composition, integrity, and compartmentation of multiphasic condensates to selectively affect the epitranscriptome.

## Methods

### Cell culture and chemical inhibitors

HEK293T was maintained in DMEM supplemented with 10% fetal bovine serum (FBS). Every 3 to 4 days, cells were trypsinized, diluted (1 to 5), and plated onto new dish to avoid high confluency. For confocal imaging, cells were plated on a 96-well glass bottom plate (P96-1.5H-N, Cellvis), which was precoated with poly-l-lysine (Sigma), and 150 ng per well of total plasmids were transfected with Lipofectamine 2000 (Life Technologies, 11668027) following manufacturer’s recommended protocol. Actinomycin D (2 to 5 μg/ml, A1410, Sigma), PF9366 (5 to 20 μM, HY-107778, MedChemExpress), and/or ADOX (40 μM, S8608, Selleckchem) were pretreated for 4 hours before imaging. DMSO was used as vehicle control. SAM (S-Adenosyl-L-methionine p-toluenesulfonate, NA04017, Carbosynth) were supplied to PF9366-pretreated cells 1 hour before imaging (3 hours after PF9366 treatment), together with PF9366. p-Toluenesulfonic acid monohydrate (Sigma) was used as vehicle control for SAM.

### Plasmids

Plasmid Cry2-mCherry was prepared by reversing the E490G mutation in Cry2olig-mCherry [34] (Addgene #60032) and used as a backbone for Cry2-mCh-METTL3 and Cry2-mCh-METTL14 constructs. cDNAs of METTL3 and METTL14 were amplified from pcDNA3/FLAG-METTL3 (Addgene #53739) and pcDNA3/FLAG-METTL14 (Addgene #53740) and C-terminally inserted into linearized Cry2-mCherry using Gibson assembly. EGFP was N-terminally inserted into pcDNA3/FLAG METTL3, METTL14 and WTAP (Addgene #53741) for coexpression and FLIM experiments. Mutant constructs were generated by using Q5 Site-Directed Mutagenesis Kit (E0554S, NEB) with following primers (uppercases indicate nucleic acid substitution intended): ΔNLS forward, 5′- catgctgcctcagatgttgatc-3′; ΔNLS reverse, 5′- ggctggctcctttgctggttc-3′; Loop to 4A forward, 5′-GCCGCCaaggaacactgcttggttggtgt-3′; Loop to 4A reverse, 5′-GGCGGCatttgtcttcacccaaataat-3′; W475A/N477A forward, 5′-ttgGCccatgggaaggaacactgctt-3′; W475A/N477A reverse, 5′-GGCgtgacctgtacggcctgtccga-3′; ΔLH forward, 5′-ctacggaatccagaggcagc-3′; ΔLH reverse, 5′-cttgtacagctcgtccatg-3′; C294A forward, 5′-GCCcgcaagctgcacttcagacga-3′; C294A reverse, 5′-gggtcgatcagcatcactgg-3′; C326A forward, 5′-GCcaagtatgttcactatgaaattgatgc-3′; C326A reverse, 5′-ggtatccatgtggaaacatgtattaagga-3′; R300D/R301D forward, 5′- GACGACattatcaataaacacactgatgagtctttaggtga-3′; R300D/R301D reverse, 5′-gaagtgcagcttgcgacagg-3′; F316A/F321A forward, 5′-cttaatacatgtGCCcacatggatacctgcaagtatgt-3′, F316A/F321A reverse, 5′-GGCagagcagtcacctaaagactcatcagtg-3′; D395A forward, 5′-gCcccaccctgggatattcaca-3′; D395A reverse, 5′-agccatcacaactgcaaacttgc-3′; N549A/Q550A forward, 5′-GCCctggatgggatccacctactaga-3′; N549A/Q550A reverse, 5′-gGCtccaagggtgatccagttgggt-3′; R508H forward, 5′- gctgaggttcAttccaccagt-3′; R508H reverse, 5′-tacgatcacatcacaatcc-3′; R415C forward, 5′-tgatgagatgTgcaggctcaa-3′; R415C reverse, 5′-tctgtcagggtcccatag-3′; E516K forward, 5′-taaaccagatAaaatctatggcatgattg-3′; E516K reverse, 5′-tgactggtggaacgaacc-3′.

### Immunocytochemistry

SH-SY5Y cells were passaged at 50,000 cells, and HeLa cells were passaged at 30,000 cells per well onto a 96-well glass bottom plate. After 1 day, cells were fixed in 2.5% w/v paraformaldehyde for 10 minutes at room temperature. This was followed by 5 minutes in 100 mM glycine to neutralize any unreacted paraformaldehyde. Next, cells were permeabilized with 0.25% Triton X-100 at room temperature for 10 minutes and washed 3× for 5 minutes each with PBS. Cells were blocked for 1 hour at room temperature with 4% BSA, 1% NGS, and 0.25% Triton X-100 in PBS. Primary antibodies against METTL3 (Abcam, ab195352) and pSC35 (Santa Cruz Biotechnology, sc-53518) were incubated overnight at 4°C, followed by 3X PBS wash. Finally, secondary antibodies and Hoescht were incubated for 1 hour at room temperature, followed by 3X PBS washes. At this point, cells were imaged in PBS buffer. For the hexanediol treatment, cells were pretreated with 1% Tween 20 in Dulbecco’s phosphate-buffered saline (DPBS) for 10 minutes at room temperature, then washed twice with DPBS. Cells were then treated with either 5% 2,5-hexanediol or 1,6-hexanediol for 5 minutes prior to fixation.

### Radial distribution analysis

Images of METTL3 and pSC35 were analyzed using a combination of ilastik [56] and MATLAB. For determining the centroids of nuclear speckles, pSC35 images were segmented into individual condensates using ilastik, and the centroids for the resulting masks were calculated in MATLAB. The radial distributions of each nuclear speckle for the METTL3 and pSC35 signals were calculated using a custom script. Final data were presented as the average of standard error mean of 67 nuclear speckles across 5 cells.

### Live-cell imaging

At 48 hours posttransfection incubation, cells were loaded into temperature- and CO2-controlled live-cell imaging chamber of Leica SP8 confocal microscope. Cells were imaged typically by use of 2 laser wavelengths (488 nm for Cry2 activation and 560 nm for mCherry imaging). Time series images for demixing index plots were taken every 2 seconds for at least 300 seconds with 488 nm illumination. FRAP assay was carried out by use of FRAP wizard in LAS-X software. Designated regions (region of interests, (ROIs)) were photobleached with UV for 60 seconds, followed by time series imaging to observe fluorescence recovery. Fluorescent intensities from ROIs were measured with ImageJ software (NIH). For live-cell DNA and RNA staining, Hoechst 33342, Trihydrochloride, Trihydrate (ThermoFisher Scientific, H3570), and SYTO RNASelect Green Fluorescent Cell Stain (ThermoFisher Scientific, S32703) was treated for 20 minutes according to manufacturer’s protocol and washed 3 times with complete media, followed by confocal imaging.

### Fluorescent lifetime imaging microscopy (FLIM)

FLIM was recorded on a Leica SP8 confocal microscope equipped with a 63× glycerol immersion objective. Confocal images from cells cotransfected with EGFP and mCherry pairs, fused to proteins of interest, were taken after blue light illumination (30 seconds) to activate Cry2 oligomerization, followed immediately by FLIM recording. EGFP lifetimes were measured using pulsed white light laser (Leica; 80,000 MHz pulsing) with excitation at 488 nm. Emission was collected with a Hybrid Detector and processed by a PicoHarp 300 Time-Corrected Single Photon Counting (TCSPC) system (PicoQuant). Photons were collected from inside condensates or nucleoplasm that lacks condensates to compare. At least 300,000 photons were recorded for each measurement and analyzed. In a control line, containing only EGFP and mCherry without fusion to any proteins, the lifetime of the donor (EGFP) was fit to a single exponential decay curve. Subsequent data were fit to a double exponential decay in which the higher, non-FRETing lifetime was treated as a constant as determined from the control experiment with the second lifetime representing the FRET component of the decay curve.

### Co-immunoprecipitation

For immunoprecipitations of tagged proteins, transfected HEK293T cells were lysed in immunoprecipitation lysis buffer (50 mM Tris–HCl (pH 7.5), 150 mM NaCl, 0.5% Triton X-100, 1 mM EDTA) with cOmplete, Mini, EDTA-free Protease Inhibitor Cocktail (Sigma). Immunoprecipitation was carried out using Pierce Anti-DYKDDDDK Magnetic Agarose (ThermoFisher Scientific, A36797) following the manufacturer’s protocol. Magnetic beads were washed 5 times with immunoprecipitation lysis buffer, and proteins were eluted with 2X Laemmli Sample Buffer for 5 minutes at 95°C (Biorad, 1610737). Proteins were resolved by SDS-PAGE, transferred onto nitrocellulose membrane, and subjected to immunoblotting using antibodies against FLAG- or HA-tag. The membranes were stained with secondary antibodies Alexa Flour 680-goat anti-mouse IgG (ThermoFisher Scientific) and Alexa Flour 790-goat anti-rabbit IgG (LI-COR) for 1 hour at room temperature. Protein bands were visualized with LI-COR Odyssey Imaging System (LI-COR).

### Statistical analysis

For condensate formation evaluation, we measured fluorescence intensity and standard deviation of ROIs and calculated coefficient of variation (CV). The minimum CV value among the time series images was subtracted from CVs at each time point to determine “demixing index” for each observation (ROIs). Condensate-forming demixing threshold is set as 0.09 to 0.11 as described in S1D Fig. To determine statistical significance of “condensate formation ability” of each given experimental condition, demixing index above the threshold area was tested using one-sample *t* test with the hypothetical value 0.11, the upper boundary of the threshold. Demixing index below the threshold area was considered as diffused phase and not subjected to statistical analysis. To compare demixing indices between samples (Fig 1A), we used Student *t* test.

## Supporting information

S1 Fig Cry2-mCh-METTL3 undergoes light-inducible LLPS. (A) Phase separation of METTL3 and pSC35 in SH-SY5Y cells. (Left) Endogenous METTL3 (green) and the nuclear speckle marker pSC35 (red) were stained using ICC. A layered and punctate distribution of METTL3 was observed surrounding central pSC35 staining. (Right) After partitioning individual nuclear speckles, the centroids of each speckle were determined. The radial distribution profiles for METTL3 and pSC35 were determined using a series of concentric circles. For pSC35, most of the fluorescent intensity quickly dies off with increase radius. In contrast, METTL3 intensity peaks at around 0.4 μM before dying off at larger radii. Scale bar = 5 μm. (B) Schematics of constructs used in this study. (C) Expression levels of Cry2 fusion proteins are represented by mCherry signal in HEK293T cells at 48 hours posttransfection of indicated plasmids. Scale bars = 10 μm. (D) Demixing index plot by normalized CV and threshold (0.9–1.1) for evaluating droplets formation. Time series confocal images were taken with HEK293T cells expressing Cry2-mCh-METTL3 with blue light (488 nm) stimulation. Scale bar = 5 μm. (E) Representative images (left) and quantitation (right) representing demixing of Cry2-mCh-METTL3 or METTL14 that are cotransfected with FLAG-METTL3, after blue light stimulation. Scale bar = 5 μm. Gray area (0.09~0.11) is set as threshold of condensate formation. (F) Predicted structural disorder probability by VSL2 algorithm (http://www.pondr.com). The underlying data for the graphs presented can be found in S1 Values For Plots. CV, coefficient of variation; ICC, immunocytochemistry; LLPS, liquid–liquid phase separation; METTL3, methyltransferase-like 3; METTL14, methyltransferase-like 14; WTAP, Wilms tumor suppressor-1–associated protein.
(TIFF)

S2 Fig Cry2-mCh-METTL3 ΔLH forms liquid condensates that lack WTAP. (A) Representative images showing localization of EGFP control, EGFP-METTL14 or EGFP-WTAP after Cry2-mCh-METTL3 ΔLH condensate formation by light stimulation. Scale bar = 5 μm. METTL3, methyltransferase-like 3; METTL14, methyltransferase-like 14; WTAP, Wilms tumor suppressor-1–associated protein.
(TIFF)

S3 Fig Cry2-mCh-METTL3 interaction with EGFP-METTL3/14. (A) Co-immunoprecipitation assay with HEK293T coexpressing Cry2-mCh-METTL3 and EGFP-tagged METTL3 or METTL14. IB, immunoblot; IP, immunoprecipitation; METTL3, methyltransferase-like 3; METTL14, methyltransferase-like 14; WCL, whole cell lysate; WTAP, Wilms tumor suppressor-1–associated protein.
(TIFF)

S4 Fig SAM binding–deficient mutants retain the ability to interact with METTL3/14. (A) Co-immunoprecipitation assay with HEK293T coexpressing Cry2-mCh-METTL3 and FLAG-tagged METTL3 WT, D395A or N549A/Q550A. (B) Co-immunoprecipitation assay to examine interaction between Cry2-mCh-METTL3 WT, D395A or N549A/Q550A and FLAG-METTL4. IB, immunoblot; IP, immunoprecipitation; METTL3, methyltransferase-like 3; METTL14, methyltransferase-like 14; SAM, S-adenosylmethionine.
(TIFF)

S1 Raw Images Original scan images for Figs 2E, S3A, S4A, and S4B. (PDF)

S1 Values For Plots Numerical values used for plots and statistical analysis in Figs 1A, 1C, 1K, 3B, 4A, 4B, 4C, 4D, 4G, 4I, 4J, S1A, S1D, S1E, and S1F. (XLSX)

## Abbreviations

ADOX: adenosine dialdehyde
CV: coefficient of variation
DPBS: Dulbecco’s phosphate-buffered saline
FBS: fetal bovine serum
FLIM: fluorescent lifetime imaging microscopy
FLIM-FRET: fluorescent lifetime imaging microscopy–Förster resonance energy transfer
FRAP: fluorescence recovery after photobleaching
ICC: immunocytochemistry
IDR: intrinsically disordered region
LH: leader helix
LLPS: liquid–liquid phase separation
MTD: methyltransferase domain
METTL3: methyltransferase-like 3
METTL14: methyltransferase-like 14
NLS: nuclear localization signal
RBP: RNA binding protein
ROI: region of interest
SAH: S-adenosylhomocysteine
SAM: S-adenosylmethionine
TCGA: The Cancer Genome Atlas
WTAP: Wilms tumor suppressor-1–associated protein
ZnF: Zinc finger

## References

[1] Jia G, Fu Y, Zhao X, Dai Q, Zheng G, Yang Y, et al. N6-Methyladenosine in nuclear RNA is a major substrate of the obesity-associated FTO. Nat Chem Biol. 2011;7:885–7. doi: 10.1038/nchembio.687 22002720 PMC3218240

[2] Liu J, Gao M, He J, Wu K, Lin S, Jin L, et al. The RNA m^6^A reader YTHDC1 silences retrotransposons and guards ES cell identity. Nature. 2021;591:322–6. doi: 10.1038/s41586-021-03313-9 33658714

[3] Wang Y, Li Y, Toth JI, Petroski MD, Zhang Z, Zhao JC. N6-methyladenosine modification destabilizes developmental regulators in embryonic stem cells. Nat Cell Biol. 2014;16:1–10. doi: 10.1038/ncb2902 24394384 PMC4640932

[4] Geula S, Moshitch-Moshkovitz S, Dominissini D, Mansour AAF, Kol N, Salmon-Divon M, et al. m^6^A mRNA methylation facilitates resolution of naïve pluripotency toward differentiation. Science. 2015;347:1002–6. doi: 10.1126/science.1261417 25569111

[5] Yoon K, Ringeling FR, Vissers C, He C, Yoon K, Ringeling FR, et al. Temporal Control of Mammalian Cortical Neurogenesis by m^6^ A Methylation. Cell. 2017;171:877–89. doi: 10.1016/j.cell.2017.09.003 28965759 PMC5679435

[6] Shi H, Zhang X, Weng Y, Lu Z, Liu Y, Lu Z, et al. m^6^A facilitates hippocampus-dependent learning and memory through YTHDF1. Nature. 2018;563:249–53. doi: 10.1038/s41586-018-0666-1 30401835 PMC6226095

[7] Koranda JL, Dore L, Koranda JL, Dore L, Shi H, Patel MJ, et al. Mettl14 Is Essential for Epitranscriptomic Regulation of Striatal Function and Learning morphological changes. Neuron. 2018;99:283–292.e5. doi: 10.1016/j.neuron.2018.06.007 30056831 PMC6082022

[8] Weng H, Huang H, Wu H, Qin X, Zhao BS, Dong L, et al. METTL14 Inhibits Hematopoietic Stem/Progenitor Differentiation and Promotes Leukemogenesis via mRNA m^6^A Modification. Cell Stem Cell. 2018;22:191–205.e9. doi: 10.1016/j.stem.2017.11.016 29290617 PMC5860916

[9] Panneerdoss S, Eedunuri VK, Yadav P, Timilsina S, Rajamanickam S, Viswanadhapalli S, et al. Cross-talk among writers, readers, and erasers of m^6^A regulates cancer growth and progression. Sci Adv. 2018;4:955–63. doi: 10.1126/sciadv.aar8263 30306128 PMC6170038

[10] Lin S, Choe J, Du P, Triboulet R, Gregory RI. The m^6^A Methyltransferase METTL3 Promotes Translation in Human Cancer Cells. Mol Cell. 2016;62:335–45. doi: 10.1016/j.molcel.2016.03.021 27117702 PMC4860043

[11] Xiao W, Adhikari S, Xiao W, Adhikari S, Dahal U, Chen Y, et al. Article Nuclear m^6^A Reader YTHDC1 Regulates mRNA Splicing. Mol Cell. 2016;61:507–19. doi: 10.1016/j.molcel.2016.01.012 26876937

[12] Shi H, Wang X, Lu Z, Zhao BS, Ma H, Hsu PJ, et al. YTHDF3 facilitates translation and decay of N6-methyladenosine-modified RNA. Nat Publ Group. 2017;27:315–28. doi: 10.1038/cr.2017.15 28106072 PMC5339834

[13] Meyer KD, Patil DP, Zhou J, Pestova TV, Qian S, Jaffrey SR, et al. 5’ UTR m^6^A Promotes Cap-Independent Translation. Cell. 2015;163:999–1010. doi: 10.1016/j.cell.2015.10.012 26593424 PMC4695625

[14] Wang X, Lu Z, Gomez A, Hon GC, Yue Y, Han D, et al. N6-methyladenosine-dependent regulation of messenger RNA stability. Nature. 2014;505:117–20. doi: 10.1038/nature12730 24284625 PMC3877715

[15] Roundtree IA, Luo G, Zhang Z, Wang X, Zhou T, Cui Y, et al. YTHDC1 mediates nuclear export of N 6—methyladenosine methylated mRNAs. Elife. 2017;6:e31311. doi: 10.7554/eLife.31311 28984244 PMC5648532

[16] Wang P, Doxtader KA, Nam Y. Structural basis for cooperative function of Mettl3 and Mettl14 methyltransferases. Mol Cell. 2016;63:306–17. doi: 10.1016/j.molcel.2016.05.041 27373337 PMC4958592

[17] Wang X, Feng J, Xue Y, Guan Z, Zhang D, Liu Z, et al. Structural basis of N6-adenosine methylation by the METTL3-METTL14 complex. Nature. 2016;534:575–8. doi: 10.1038/nature18298 27281194

[18] Śledź P, Jinek M. Structural insights into the molecular mechanism of the m^6^A writer complex. Elife. 2016. doi: 10.7554/eLife.18434 27627798 PMC5023411

[19] Huang J, Dong X, Gong Z, Qin L, Yang S, Zhu Y, et al. Solution structure of the RNA recognition domain of METTL3-METTL14 N 6 -methyladenosine methyltransferase. Protein Cell. 2019;10:272–84. doi: 10.1007/s13238-018-0518-7 29542011 PMC6418081

[20] Liu J, Yue Y, Han D, Wang X, Fu Y, Zhang L, et al. A METTL3 –METTL14 complex mediates mammalian nuclear RNA N6-adenosine methylation. Nat Chem Biol. 2014;10:2013–5. doi: 10.1038/nchembio.1432 24316715 PMC3911877

[21] Ping XL, Sun BF, Wang L, Xiao W, Yang X, Wang WJ, et al. Mammalian WTAP is a regulatory subunit of the RNA N6-methyladenosine methyltransferase. Cell Res. 2014;24:177–89. doi: 10.1038/cr.2014.3 24407421 PMC3915904

[22] Decker CJ, Parker R. P-bodies and stress granules: Possible roles in the control of translation and mRNA degradation. Cold Spring Harb Perspect Biol. 2012;4:a012286. doi: 10.1101/cshperspect.a012286 22763747 PMC3428773

[23] Brangwynne CP, Mitchison TJ, Hyman AA. Active liquid-like behavior of nucleoli determines their size and shape in Xenopus laevis oocytes. Proc Natl Acad Sci U S A. 2011;108:4334–9. doi: 10.1073/pnas.1017150108 21368180 PMC3060270

[24] Brangwynne CP. Soft active aggregates: Mechanics, dynamics and self-assembly of liquid-like intracellular protein bodies. Soft Matter. 2011;7:3052–9. doi: 10.1039/c0sm00981d

[25] Lamond AI, Spector DL. Nuclear speckles: A model for nuclear organelles. Nat Rev Mol Cell Biol. 2003;4:605–12. doi: 10.1038/nrm1172 12923522

[26] Buchan JR, Parker R. Eukaryotic Stress Granules: The Ins and Outs of Translation. Mol Cell. 2009;36:932–41. doi: 10.1016/j.molcel.2009.11.020 20064460 PMC2813218

[27] Anderson P, Kedersha N. RNA granules: Post-transcriptional and epigenetic modulators of gene expression. Nat Rev Mol Cell Biol. 2009;10:430–6. doi: 10.1038/nrm2694 19461665

[28] Laflamme G, Mekhail K. Biomolecular condensates as arbiters of biochemical reactions inside the nucleus. Commun Biol. 2020;3:1–8. doi: 10.1038/s42003-019-0734-6 33319830 PMC7738674

[29] Hyman AA, Weber CA, Frank J. Liquid-Liquid Phase Separation in Biology. Annu Rev Cell Dev Biol. 2014;30:39–58. doi: 10.1146/annurev-cellbio-100913-013325 25288112

[30] Fei J, Jadaliha M, Harmon TS, Li ITS, Hua B, Hao Q, et al. Quantitative analysis of multilayer organization of proteins and RNA in nuclear speckles at super resolution. J Cell Sci. 2017;130:4180–92. doi: 10.1242/jcs.206854 29133588 PMC5769577

[31] Li P, Banjade S, Cheng HC, Kim S, Chen B, Guo L, et al. Phase transitions in the assembly of multivalent signalling proteins. Nature. 2012;483:336–40. doi: 10.1038/nature10879 22398450 PMC3343696

[32] Uversky VN. Intrinsically disordered proteins in overcrowded milieu: Membrane-less organelles, phase separation, and intrinsic disorder. Curr Opin Struct Biol. 2017;44:18–30. doi: 10.1016/j.sbi.2016.10.015 27838525

[33] Shin Y, Berry J, Pannucci N, Haataja MP, Toettcher JE, Brangwynne CP, et al. Spatiotemporal Control of Intracellular Phase Transitions Using Light-Activated optoDroplets. Cell. 2017;168:159–71. doi: 10.1016/j.cell.2016.11.054 28041848 PMC5562165

[34] Taslimi A, Vrana JD, Chen D, Borinskaya S, Mayer BJ, Kennedy MJ, et al. An optimized optogenetic clustering tool for probing protein interaction and function. Nat Commun. 2014;5:1–9. doi: 10.1038/ncomms5925 25233328 PMC4170572

[35] Zhu T, Roundtree IA, Wang P, Wang X, Wang L, Sun C, et al. Crystal structure of the YTH domain of YTHDF2 reveals mechanism for recognition of N6-methyladenosine. Cell Res. 2014;24:1493–6. doi: 10.1038/cr.2014.152 25412661 PMC4260350

[36] Fu Y, Zhuang X. m^6^A-binding YTHDF proteins promote stress granule formation. Nat Chem Biol. 2020;16:955–63. doi: 10.1038/s41589-020-0524-y 32451507 PMC7442727

[37] Ries RJ, Zaccara S, Klein P, Olarerin-George A, Namkoong S, Pickering BF, et al. m^6^A enhances the phase separation potential of mRNA. Nature. 2019;571:424–8. doi: 10.1038/s41586-019-1374-1 31292544 PMC6662915

[38] Wang J, Wang L, Diao J, Shi YG, Shi Y, Ma H, et al. Binding to m^6^A RNA promotes YTHDF2-mediated phase separation. Protein Cell. 2020;11:304–7. doi: 10.1007/s13238-019-00660-2 31642031 PMC7093369

[39] Bokar JA, Shambaugh ME, Polayes D, Matera AG, Rottman FM. Purification and cDNA cloning of the AdoMet-binding subunit of the human mRNA (N6-adenosine)-methyltransferase. RNA. 1997;3:1233–47. 9409616 PMC1369564

[40] Conicella AE, Zerze GH, Mittal J, Fawzi NL. ALS mutations disrupt phase separation mediated by α-helical structure in the TDP-43 low-complexity C-terminal domain. Structure. 2016;24:1537–49. doi: 10.1016/j.str.2016.07.007 27545621 PMC5014597

[41] Lin Y, Protter DSW, Rosen MK, Parker R. Formation and maturation of phase-separated liquid droplets by RNA-binding proteins. Mol Cell. 2015;60:208–19. doi: 10.1016/j.molcel.2015.08.018 26412307 PMC4609299

[42] Molliex A, Temirov J, Lee J, Coughlin M, Kanagaraj AP, Kim HJ, et al. Phase separation by low complexity domains promotes stress granule assembly and drives pathological fibrillization. Cell. 2015;163:123–33. doi: 10.1016/j.cell.2015.09.015 26406374 PMC5149108

[43] Bugaj LJ, Choksi AT, Mesuda CK, Kane RS, Schaffer DV. Optogenetic protein clustering and signaling activation in mammalian cells. Nat Methods. 2013;10:249–52. doi: 10.1038/nmeth.2360 23377377

[44] Sabari BR, Dall’Agnese A, Boija A, Klein IA, Coffey EL, Shrinivas K, et al. Coactivator condensation at super-enhancers links phase separation and gene control. Science. 2018;361. doi: 10.1126/science.aar3958 29930091 PMC6092193

[45] Huang H, Weng H, Zhou K, Wu T, Zhao BS, Sun M, et al. Histone H3 trimethylation at lysine 36 guides m^6^A RNA modification co-transcriptionally. Nature. 2019;567:414–9. doi: 10.1038/s41586-019-1016-7 30867593 PMC6438714

[46] Schöller EVA, Weichmann F, Treiber T, Ringle SAM, Treiber N, Flatley A, et al. Interactions, localization, and phosphorylation of the m^6^A generating METTL3–METTL14–WTAP complex. RNA. 2018;24:499–512. doi: 10.1261/rna.064063.117 29348140 PMC5855951

[47] Meyer KD, Saletore Y, Zumbo P, Elemento O, Mason CE, Jaffrey SR. Comprehensive analysis of mRNA methylation reveals enrichment in 3′ UTRs and near stop codons. Cell. 2012;149:1635–46. doi: 10.1016/j.cell.2012.05.003 22608085 PMC3383396

[48] Llères D, Swift S, Lamond AI. Detecting protein-protein interactions in vivo with FRET using multiphoton fluorescence lifetime imaging microscopy (FLIM). Curr Protoc Cytom. 2007;Chapter 12: 1–19. doi: 10.1002/0471142956.cy0101s39 18770849

[49] Akrap N, Seidel T, Barisas BG. Förster distances for fluorescence resonant energy transfer between mCherry and other visible fluorescent proteins. Anal Biochem. 2010;402:105–6. doi: 10.1016/j.ab.2010.03.026 20347671 PMC2885848

[50] Quinlan CL, Kaiser SE, Bolaños B, Nowlin D, Grantner R, Karlicek-bryant S, et al. Targeting S-adenosylmethionine biosynthesis with a novel allosteric inhibitor of Mat2A. Nat Chem Biol. 2017;13:785–92. doi: 10.1038/nchembio.2384 28553945

[51] Castro R, Rivera I, Blom HJ, Jakobs C, Tavares de Almeida I. Homocysteine metabolism, hyperhomocysteinaemia and vascular disease: An overview. J Inherit Metab Dis. 2006;29:3–20. doi: 10.1007/s10545-006-0106-5 16601863

[52] Bindu Madhavan GV, McGee DPC, Rydzewski RM, Boehme R, Martin JC, Prisbe EJ. Synthesis and antiviral evaluation of 6′-substituted aristeromycins: potential mechanism-based inhibitors of S-adenosylhomocysteine hydrolase. J Med Chem. 1988;31:1798–804. doi: 10.1021/jm00117a021 2842505

[53] Agarwala SD, Blitzblau HG, Hochwagen A, Fink GR. RNA methylation by the MIS complex regulates a cell fate decision in yeast. PLoS Genet. 2012;8:1–13. doi: 10.1371/journal.pgen.1002732 22685417 PMC3369947

[54] Sanders DW, Kedersha N, Lee DSW, Strom AR, Drake V, Riback JA, et al. Competing protein-RNA interaction networks control multiphase intracellular organization. Cell. 2020;181:306–324.e28. doi: 10.1016/j.cell.2020.03.050 32302570 PMC7816278

[55] Wang X, Jiang B, Gu L, Chen Y, Mora M, Zhu M, et al. A photoregulatory mechanism of the circadian clock in Arabidopsis. Nature Plants. 2021;7:1397–408. doi: 10.1038/s41477-021-01002-z 34650267

[56] Berg S, Kutra D, Kroeger T, Straehle CN, Kausler BX, Haubold C, et al. Ilastik: Interactive Machine Learning for (Bio)Image Analysis. Nat Methods. 2019;16:1226–32. doi: 10.1038/s41592-019-0582-9 31570887

