# Supplementary figures and images for: Dynamic assembly of the mRNA m6A methyltransferase complex is regulated by METTL3 phase separation
Source: PLoS Biol. 2022 Feb 10;20(2):e3001535. doi: 10.1371/journal.pbio.3001535 (PMC8865655; doi:10.1371/journal.pbio.3001535)

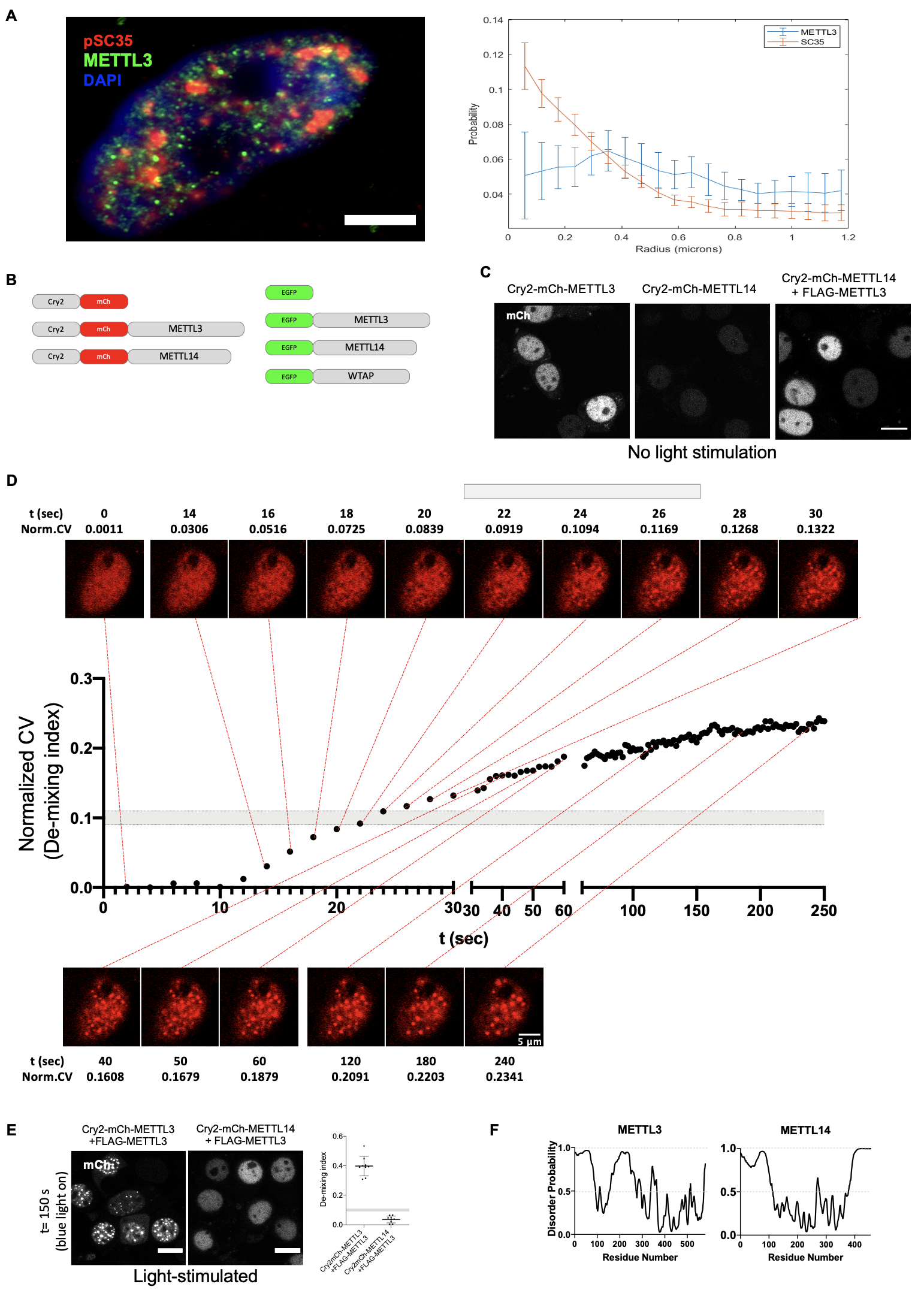

Supplement: S1 Fig — (A) Phase separation of METTL3 and pSC35 in SH-SY5Y cells. (Left) Endogenous METTL3 (green) and the nuclear speckle marker pSC35 (red) were stained using ICC. A layered and punctate distribution of METTL3 was observed surrounding central pSC35 staining. (Right) After partitioning individual nuclear speckles, the centroids of each speckle were determined. The radial distribution profiles for METTL3 and pSC35 were determined using a series of concentric circles. For pSC35, most of the fluorescent intensity quickly dies off with increase radius. In contrast, METTL3 intensity peaks at around 0.4 μM before dying off at larger radii. Scale bar = 5 μm. (B) Schematics of constructs used in this study. (C) Expression levels of Cry2 fusion proteins are represented by mCherry signal in HEK293T cells at 48 hours posttransfection of indicated plasmids. Scale bars = 10 μm. (D) Demixing index plot by normalized CV and threshold (0.9–1.1) for evaluating droplets formation. Time series confocal images were taken with HEK293T cells expressing Cry2-mCh-METTL3 with blue light (488 nm) stimulation. Scale bar = 5 μm. (E) Representative images (left) and quantitation (right) representing demixing of Cry2-mCh-METTL3 or METTL14 that are cotransfected with FLAG-METTL3, after blue light stimulation. Scale bar = 5 μm. Gray area (0.09~0.11) is set as threshold of condensate formation. (F) Predicted structural disorder probability by VSL2 algorithm (http://www.pondr.com). The underlying data for the graphs presented can be found in S1 Values For Plots. CV, coefficient of variation; ICC, immunocytochemistry; LLPS, liquid–liquid phase separation; METTL3, methyltransferase-like 3; METTL14, methyltransferase-like 14; WTAP, Wilms tumor suppressor-1–associated protein. (TIFF) [file pbio.3001535.s001.tiff]

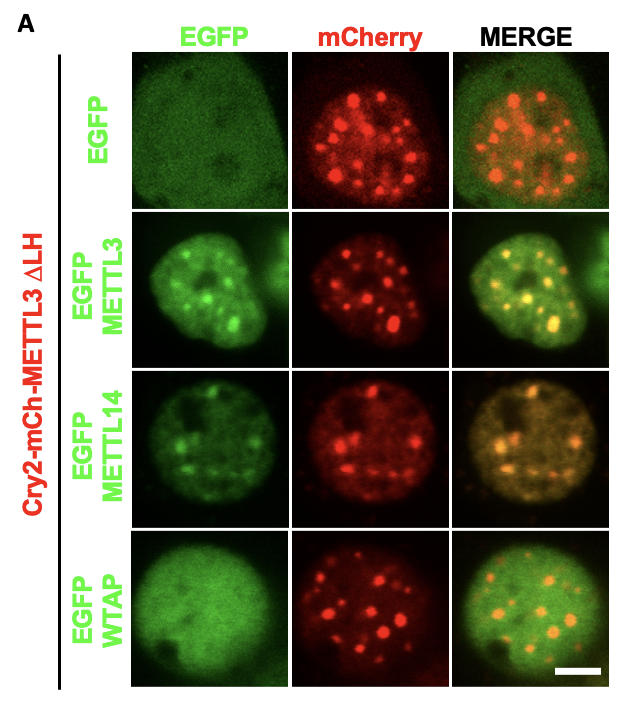

Supplement: S2 Fig — (A) Representative images showing localization of EGFP control, EGFP-METTL14 or EGFP-WTAP after Cry2-mCh-METTL3 ΔLH condensate formation by light stimulation. Scale bar = 5 μm. METTL3, methyltransferase-like 3; METTL14, methyltransferase-like 14; WTAP, Wilms tumor suppressor-1–associated protein. (TIFF) [file pbio.3001535.s002.tiff]

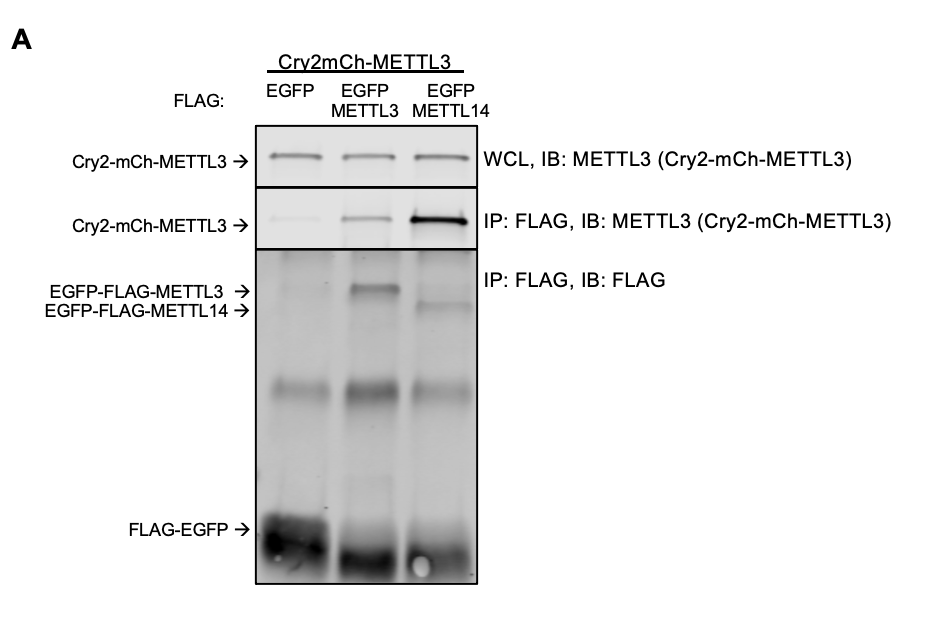

Supplement: S3 Fig — (A) Co-immunoprecipitation assay with HEK293T coexpressing Cry2-mCh-METTL3 and EGFP-tagged METTL3 or METTL14. IB, immunoblot; IP, immunoprecipitation; METTL3, methyltransferase-like 3; METTL14, methyltransferase-like 14; WCL, whole cell lysate; WTAP, Wilms tumor suppressor-1–associated protein. (TIFF) [file pbio.3001535.s003.tiff]

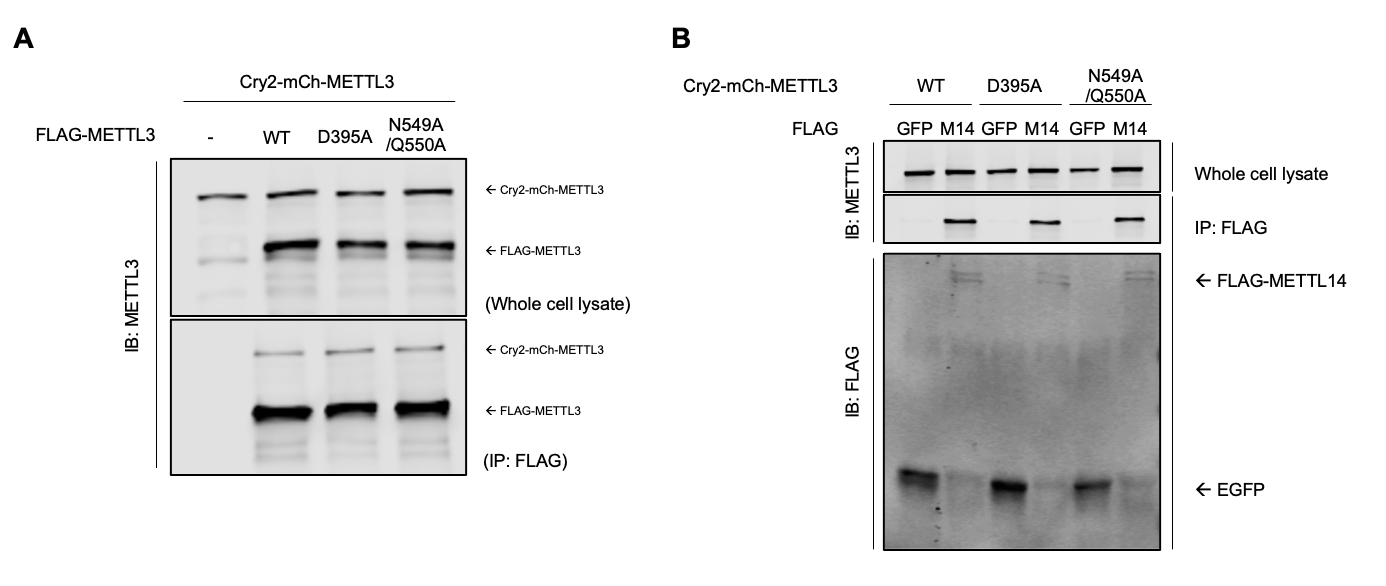

Supplement: S4 Fig — (A) Co-immunoprecipitation assay with HEK293T coexpressing Cry2-mCh-METTL3 and FLAG-tagged METTL3 WT, D395A or N549A/Q550A. (B) Co-immunoprecipitation assay to examine interaction between Cry2-mCh-METTL3 WT, D395A or N549A/Q550A and FLAG-METTL4. IB, immunoblot; IP, immunoprecipitation; METTL3, methyltransferase-like 3; METTL14, methyltransferase-like 14; SAM, S-adenosylmethionine. (TIFF) [file pbio.3001535.s004.tiff]
