# Supplementary material for: Dynamic assembly of the mRNA m6A methyltransferase complex is regulated by METTL3 phase separation
Source: PLoS Biol. 2022 Feb 10;20(2):e3001535. doi: 10.1371/journal.pbio.3001535 (PMC8865655; doi:10.1371/journal.pbio.3001535)
Supplement: S1 Raw Images — (PDF) [file pbio.3001535.s005.pdf]

Original images (blots) of Fig. 2E

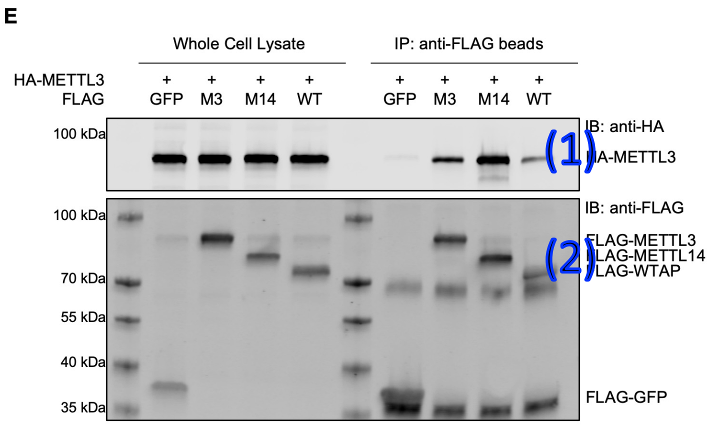

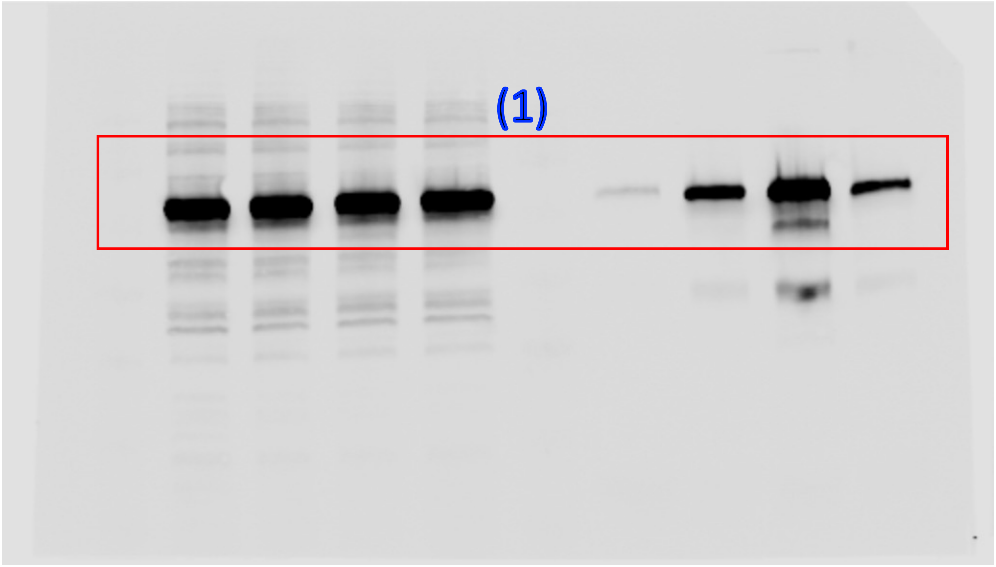

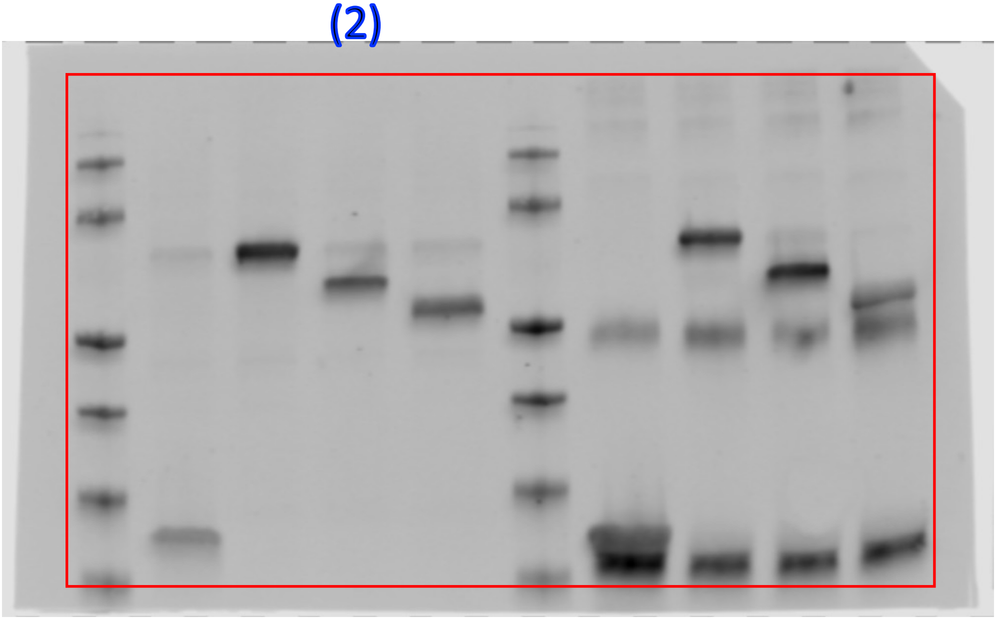

Original scans from Li-cor scanner

Original images (blots) of S3A Fig

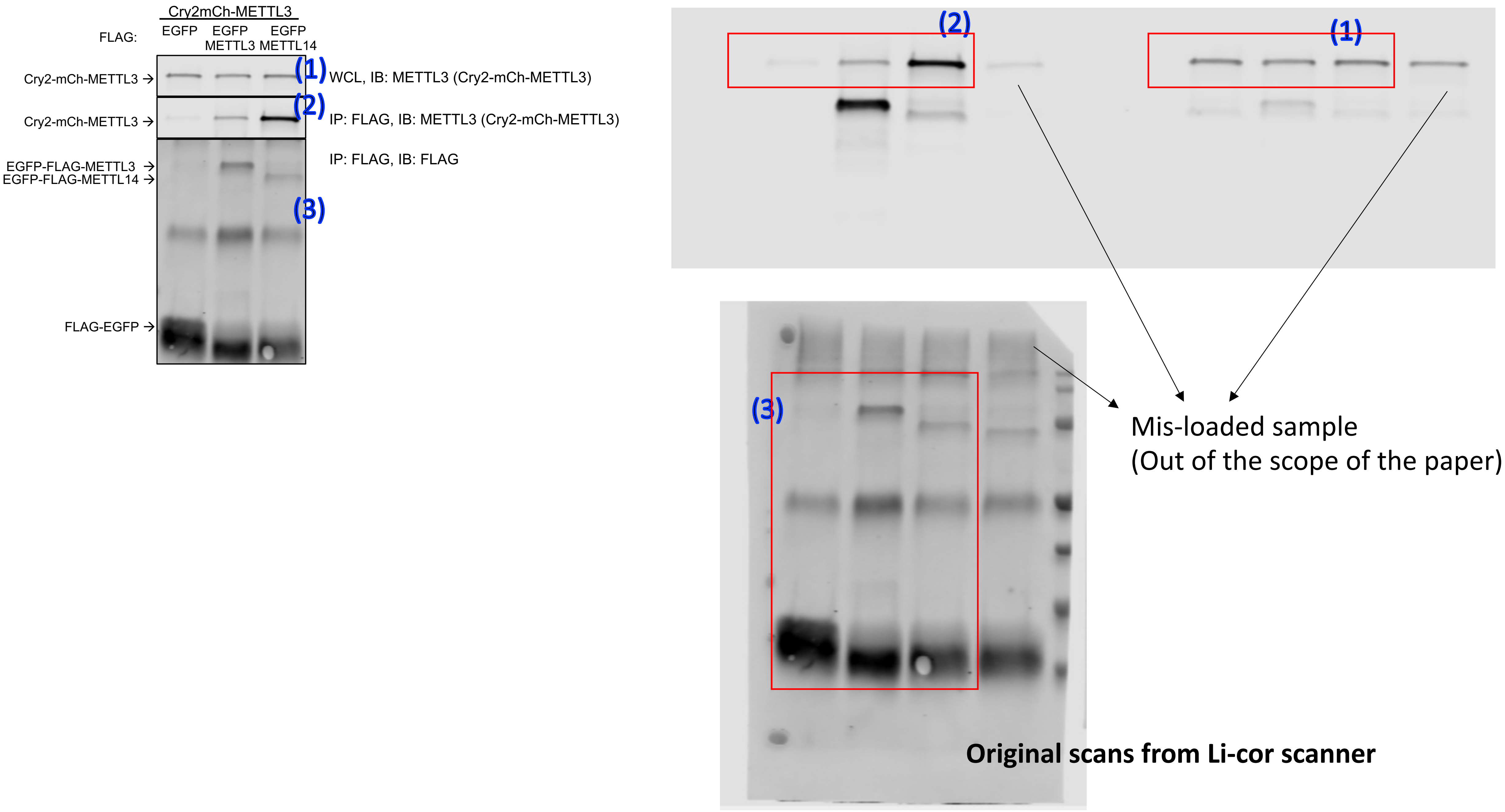

Original images (blots) of S4A and S4B Figs

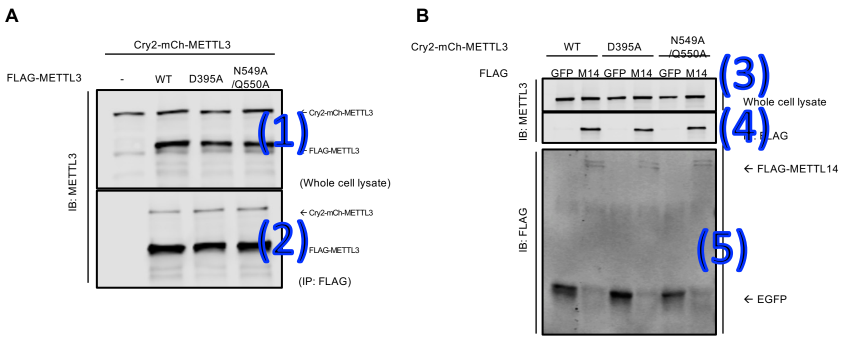

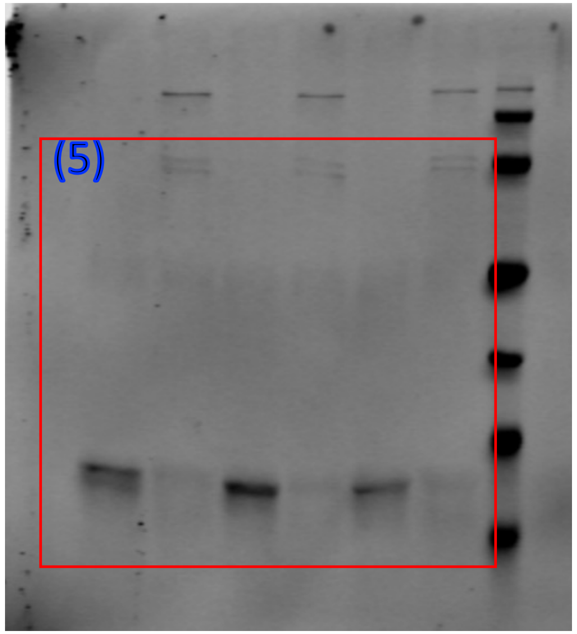

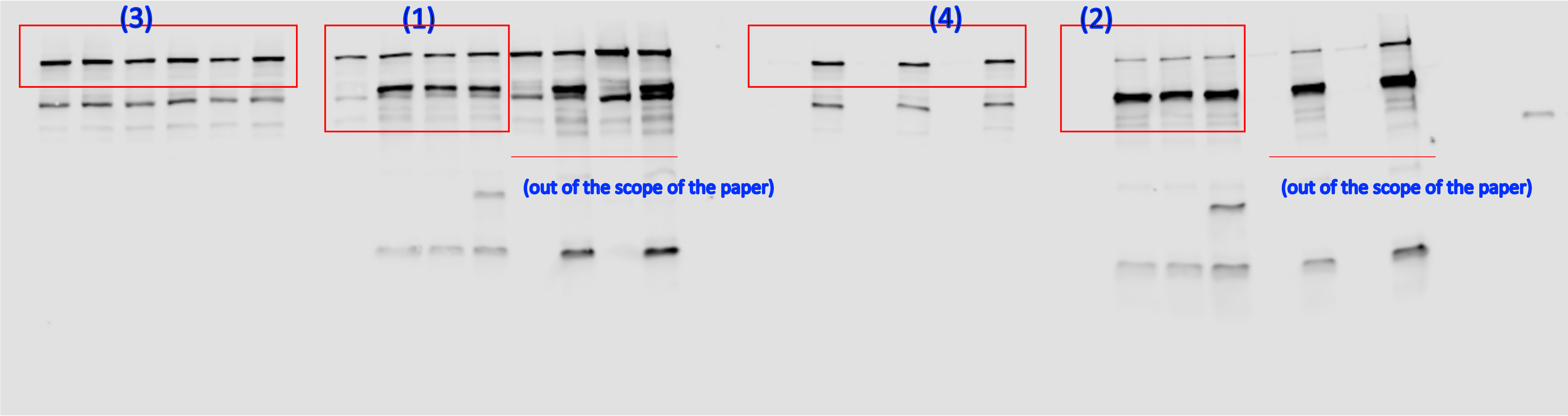

Original scans from Li-cor scanner
